# An *In Vitro* Investigation of 5-Aminolevulinic Acid Mediated Photodynamic Therapy in Bone Sarcoma

**DOI:** 10.32604/or.2025.069781

**Published:** 2026-01-19

**Authors:** Rebecca H. Maggs, Marcus J. Brookes, Kenneth S. Rankin

**Affiliations:** Translational and Clinical Research Institute, Newcastle University, Newcastle Upon Tyne, NE1 7RU, UK

**Keywords:** Bone sarcoma, photodynamic therapy (PDT), 5-aminolevulinic acid (5-ALA), osteosarcoma, chondrosarcoma, Ewing sarcoma

## Abstract

**Background:**

Photodynamic therapy (PDT) may eradicate residual malignant cells following sarcoma resection, through reactive oxygen species (ROS) mediated cytotoxicity, thus improve clinical outcomes. This study aims to assess the efficacy of 5-aminolevulinic acid (5-ALA) as a photosensitizer in combination with red light (RL) for PDT of bone sarcoma cells *in vitro*.

**Methods:**

Three bone sarcoma cell lines underwent treatment with 5-ALA and RL or sham-RL (SL). 5-ALA uptake was assessed using flow cytometry. Production of ROS was measured using CellROX Green staining and fluorescence microscopy. Cell viability was assessed using Cell Counting Kit-8 assays.

**Results:**

All cell lines showed significant 5-ALA uptake in comparison to the 0 mM control (*p* < 0.05). Production of ROS was significantly increased in cells treated with 5-ALA and RL, compared to those treated with RL and no 5-ALA or SL (*p* < 0.05). Viability was significantly reduced in cells treated with 5-ALA and RL, compared to SL (*p* < 0.05). At 72 h post-treatment, cell viability ranged from 6%–12% in 0.5 mM 5-ALA and RL-treated cells vs. 90%–137% in 0.5 mM 5-ALA and SL-treated cells.

**Conclusion:**

5-ALA-based PDT led to the desired increased production of ROS and reduction in cell viability in all cell lines. These preliminary *in vitro* results warrant further study with multicellular spheroid or animal models and suggest PDT has potential to be used as an adjuvant therapy to surgical resection in sarcoma management.

## 1 Introduction

Bone sarcomas are rare, malignant neoplasms, arising from mesenchymal cells and account for ~0.2% of cancer diagnoses [1,2]. Bone sarcomas are broadly categorised into chondrosarcoma, osteosarcoma, Ewing sarcoma or chordoma, before being divided into further subgroups based on histology [3]. The most commonly diagnosed bone sarcoma in the United Kingdom (UK) is chondrosarcoma, followed by osteosarcoma, Ewing sarcoma and chordoma, with 5-year survival rates of ~80%, ~60%, ~50% and ~65%, respectively [4]. Chondrosarcoma and chordoma are more commonly seen in adults over the age of 40 years, whereas osteosarcoma and Ewing sarcoma are more prevalent in adolescents and young adults, with a peak incidence between 10–24 years [3].

Despite the heterogeneity of bone sarcomas, general principles of treatment remain consistent [4]. Most commonly, surgical resections with wide margins are performed, combined with neoadjuvant or adjuvant chemotherapy or radiotherapy, depending on the subtype [5–7]. Surgical resection aims to remove the tumour on a macroscopic and microscopic level, achieving negative margins [8]. As such, surgical resection is more likely to induce a cure when lesions are small, not invading critical structures and access is uncomplicated, allowing resections with wide, therefore likely negative, margins [9,10]. Adjuvant therapies are further limited by the development of drug resistance and toxicity [11,12]. The importance of achieving negative margins to improve outcomes remains consistent across subtypes of bone sarcoma. Several studies have identified positive margins as an independent factor contributing towards significantly increased risk of local recurrence (LR) and eventual disease-associated death in osteosarcoma patients [13,14]. The importance of resection margins can also be seen in chondrosarcoma. Achieving wide surgical margins in dedifferentiated chondrosarcoma is recognised as necessary for optimal management, giving the highest chance of disease-free survival [15–17]. Further reports state the benefits of negative margins in Ewing sarcoma, improving local control and reducing rates of relapse [18,19].

Achieving negative margins can mean excessive resection of normal tissue, preventing adequate limb reconstruction and recovery of function [20]. As the cornerstone of sarcoma treatment, it is critical surgical resections are optimised, in terms of associated morbidity and oncological outcomes. Efforts to address this issue are currently being made using computer navigated surgery [21] and fluorescence-guided surgery (FGS) [22], a means of visualising tumour margins intraoperatively using fluorophores to guide the surgeon. SarcoSIGHT is the inaugural trial assessing the effects of FGS on the rate of positive resection margins in sarcoma [23]. The use of similar fluorophores, such as 5-aminolevulinic acid (5-ALA), could be used to visualise tumours, kill residual malignant cells and clear potential positive margins. This study explores the use of photodynamic therapy (PDT) for the treatment of sarcoma *in vitro*. This commences work towards potential use of adjunctive, intraoperative PDT for the clearance of inadvertent positive margins.

Successful PDT has three fundamental facets: a photosensitizer (PS), oxygen and an excitation light source [24]. The correct combination of these three units results in a dynamic interaction, producing reactive oxygen species (ROS) and subsequently causing cell death via a range of mechanisms [25]. The cytotoxicity resulting from this interaction means PDT has a promising role in the treatment of a broad range of malignancies [26,27]. Literature commonly considers three direct, PDT-induced mechanisms of cell death: apoptosis, autophagy and necrosis [24,25,28–30]. Indirect cell death via destruction of tumour microvasculature [29,31] and initiation of an anti-tumour immune response [29,31,32] are two further significant mechanisms. This is advantageous over some cytotoxic agents, which only trigger apoptotic death. Long-term tumour control following PDT may be explained by the amalgamation of direct, local and immunostimulatory mechanisms of cell death (e.g., release of damage-associated molecular patterns and dendritic cell activation [33]) leading to acute tumour destruction and ongoing systemic anti-tumour immunity.

PSs preferentially accumulate in tumour cells during the drug-light interval (the time between PS administration and irradiation) [25,31]. Selectivity is enhanced by the short half-lives and limited destruction range (20 nm from formation site) of ROS. The selective nature of PS uptake is highly advantageous in limiting systemic toxicity, often seen with more traditional drug-based therapeutics, such as chemotherapy. PDT can, however, still be used in combination with surgery, chemotherapy and radiotherapy, due to its lack of systemic interference, exemplifying its potential as an adjuvant therapy for sarcoma.

5-ALA is a non-proteogenic amino acid and a second-generation, exogenous PS in prodrug form [30,34,35]. It has been shown to preferentially accumulate in malignant cells compared to non-malignant cells [36,37]. Following mitochondrial accumulation, 5-ALA is metabolised to the endogenous PS: protoporphyrin IX (PpIX) [30,35], which can then be exploited for therapeutic use. In 5-ALA induced PDT (5-ALA-PDT), 630–635 nm red light (RL) excitation is frequently used [38–40]. Despite having a smaller absorption peak than blue-violet light, centred around 405 nm [38,41], irradiating cells at 630–635 nm enables adequate excitation for ROS production, whilst achieving sufficient tissue penetration due to its longer wavelength. When used intraoperatively for sarcoma, the light source could be applied directly to the wound bed, with RL penetrating and exciting the PS at greater depths than blue light, theoretically increasing chances of tumour deposit destruction, vascular damage and immuno-stimulation.

5-ALA-PDT has become established for the treatment of both benign and malignant dermatological conditions [42,43]. Research using 5-ALA in other fields has since exponentially progressed [44], including 5-ALA-PDT for oesophageal malignancy [45], hepatocellular carcinoma [27] and, namely, glioma. In glioma, 5-ALA is widely reported for PDT [30,46,47] and FDA approved for FGS [48–50]. Successful clinical trials have reported survival advantages offered by 5-ALA-PDT [51,52], evidencing the potential of 5-ALA-PDT to gain approval and aid in treating aggressive malignancies, including sarcoma. 5-ALA-PDT for the treatment of sarcoma remains a developing concept but has shown promise. Multiple studies evidence uptake and 5-ALA-PDT-induced cytotoxicity *in vitro* in sarcoma cell lines [39,40,53,54].

The main aim of this study was to assess the cellular uptake of 5-ALA, as well as the photodynamic and cytotoxic properties of RL irradiated 5-ALA against bone sarcoma cells *in vitro*. This has the long-term, translational goal of integrating intraoperative PDT with FGS at the time of sarcoma resection to clear potential positive margins, locoregional micrometastases or distant metastases.

## 2 Materials and Methods

### 2.1 Cell Lines and Culture

HT1080 (dedifferentiated chondrosarcoma, ATCC, CCL-121), U2OS (osteosarcoma; ATCC, HTB-96) and TC71 (Ewing sarcoma, ATCC, DSMZ ACC-516) cell lines were obtained from the American Type Culture Collection (ATCC; Mannassas, VA, USA), where they were authenticated by short tandem repeat profiling and were confirmed mycoplasma-free by the supplier, as well as routinely testing negative for mycoplasma contamination using polymerase chain reaction-based assays in the laboratory. The cells were cultured in RPMI-1640 Medium (Sigma-Aldrich, R8758, St. Louis, MO, USA), supplemented with 10% Foetal Bovine Serum (FBS; Gibco, Thermo Fisher Scientific, 10270-106, Waltham, MA, USA) and 1% Streptomycin/Penicillin (100 U/mL penicillin, 100 μg/mL streptomycin; Gibco, Thermo Fisher Scientific, 15140-122, Waltham, MA, USA). Cells were maintained in a humidified incubator at 37°C and 5% CO_2_. Culture medium (CM) was changed to serum-free medium (SFM) overnight before uptake and viability experiments, minimising PpIX excretion due to the FBS-containing CM [55,56]. For ROS experiments, CM was not changed to SFM overnight, minimising serum starvation, avoiding stress-induced and PDT-independent ROS production, thus false positive results [57].

### 2.2 5-ALA Preparation

5-ALA hydrochloride (Sigma-Aldrich, A7793, St. Louis, MO, USA) was dissolved in SFM to make a 100 mM (16,778.5 μg/mL) stock solution. Aliquots of stock solution were stored at −20°C until use. Serial dilutions using SFM produced 4 mM (670.4 μg/mL), 2 mM (335.2 μg/mL), 1 mM (167.6 μg/mL) and 0.5 mM (83.8 μg/mL) 5-ALA solutions, selected based on existing literature [58–60].

### 2.3 Flow Cytometry

**Figure 1:**
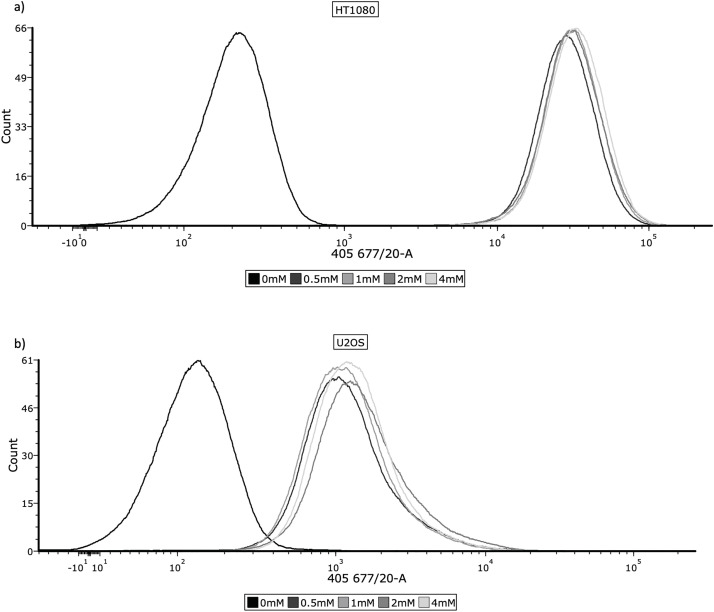

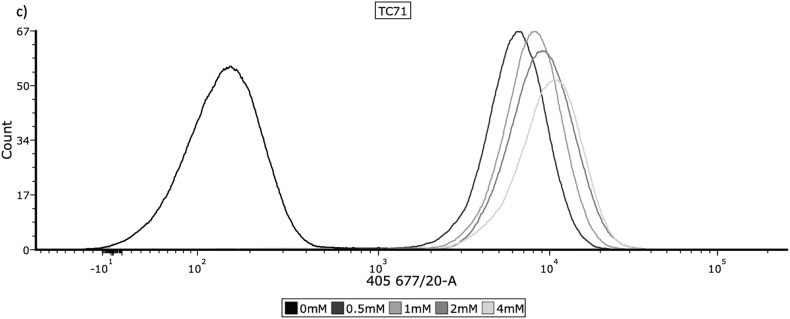
Cellular uptake of 5-ALA across the panel of cell lines. Cells were prepared for flow cytometry and analysed by a BD FACSymphony™ A5 Cell Analyzer, using the 405, 677/20 laser to measure fluorescence. The frequency histograms show fluorescence intensity, with one representative repeat of overall mean FI per concentration being plotted. The *x*-axis represents the signal intensity (biexponential scale) caused by detection of PpIX and the *y*-axis represents cell count. 10,000 events were recorded for each concentration during all 3 repeats; cell populations were gated to remove cellular debris and doublets. (**a**) HT1080, (**b**) U2OS, (**c**) TC71

HT1080, U2OS and TC71 cell lines were incubated with 0, 0.5, 1, 2 and 4 mM 5-ALA for 4 h. 5-ALA was then removed and cells were washed with PBS (pH 7.4, 1x), before being lifted with flow buffer (FB) (PBS + 5% 0.2 mM EDTA (Sigma-Aldrich, E988, St. Louis, MO, USA) + 5% BSA (Miltenyi Biotec (MACS), 130091376, Bergisch Gladbach, Germany)). Cells were resuspended in FB and ~1 × 10^6^ cells were transferred to FACS tubes, before being fixed in 3.7% formaldehyde. Cells were then washed with PBS, resuspended in FB and refrigerated in the dark. Accumulation of PpIX was detected by flow cytometry (band-pass 677/20 nm) on a BD FACSymphony™ A5 Cell Analyzer (BD Biosciences, San Jose, CA, USA). Three biological replicates were performed for each concentration. Flow cytometry data were analysed using FCS Express 7 (version 7.22.0006, *De Novo* Software, Los Angeles, CA, USA). Photomultiplier tube voltages were set identically across all groups. Single cells were gated based on forward and side scatter to exclude debris and doublets. 10,000 events were recorded for each sample. Samples incubated with 0 mM 5-ALA served as negative controls. Representative histograms and overlays are shown in Fig. 1a–c. Mean fluorescence intensity (FI) was used for statistical analysis.

### 2.4 Irradiation

Continuous wave 630 nm RL was delivered at a dosage of 20 J/cm^2^ by the LED-X LED Array (Green Leaf Scientific, Dublin, Ireland). The power output of the RL was 20 mw, the fluence rate was 75.8 mW/cm^2^ and the total exposure time was 264 s. The RL source has 96 LEDs designed specifically for uniform delivery to each well in a 96-well plate, which sits directly on top of the source. Untreated cells were exposed to sham-RL (SL) for an equal exposure time of 264 s, using the same setup without delivering biologically active 630 nm light.

### 2.5 ROS Studies

HT1080, U2OS and TC71 cell lines were seeded at a density of ~3 × 10^4^ cells/well in fibronectin-coated μCLEAR® 96-well plates (Greiner bio-one, 655097, Kremsmünster, Austria). The following day, cells were incubated with 0, 0.5, 1, 2 or 4 mM 5-ALA for 4 h. 5-ALA was then removed, cells were washed with PBS (pH 7.4, 1x) and CM was replaced. The treatment group was irradiated with RL, the control group was left in SL for an equal length of time. CM was removed and 100 μL/well of 5μM CellROX Green (Invitrogen, C10444, Waltham, MA, USA) was added and incubated for 30 min. CellROX Green was removed, cells were washed with PBS and then fixed in 3.7% formaldehyde. Cells were then washed with PBS and 100 μL/well of 1 μg/mL DAPI (Thermo Fisher Scientific, 62248, Waltham, MA, USA) was added for 15 min, before washing with and leaving in PBS. CellROX Green displays increased fluorescence after sequential oxidation by ROS and binding to nuclear or cytoplasmic DNA, facilitating analysis of increased ROS production following 5-ALA-PDT. Imaging was performed the same day using a confocal ZEISS Celldiscoverer 7 microscope (Carl Zeiss, Oberkochen, Germany) (20× magnification). Three biological replicates were performed for all cell lines at all treatment conditions. ZEISS ZEN 3.1 (blue edition) (Carl Zeiss, Oberkochen, Germany) was used for analysis and calculation of CellROX Green fluorescence intensity. Fluorescence intensities were calculated using zones of influence and sizing strategies to detect nuclei through DAPI, then measuring and providing fluorescence intensity of CellROX Green for each individual cell in that image. Median fluorescence intensities (MFIs) were used for further statistical analysis. When displayed graphically, bars represent MFI and error bars represent the interquartile range (IQR), calculated from the distribution of measurements across replicates.

### 2.6 Cell Viability Assays

HT1080, U2OS and TC71 cell lines were seeded at a density of ~3 × 10^4^ cells/well in fibronectin coated Costar® 96-well plates (Corning, 3598, Corning, NY, USA). Cells were incubated with 0, 0.5, 1, 2 or 4 mM 5-ALA for 4 h. 5-ALA was then removed, cells were washed with PBS (pH 7.4, 1x) and CM was replaced. The treatment group was irradiated with RL, the control group was left in SL for an equal length of time. Cell viability was measured at 0, 24, 48 and 72 h post-treatment with a cell counting kit-8 (CCK-8) assay (Dojindo Laboratories, CK04, Kumamoto, Japan). At each time point, 110 μL of diluted CCK-8 solution, in CM 1:10, was added to each well and incubated for 1 h. Absorbance was measured at 450 nm using a FLUOstar Omega Microplate Reader (FLUOstar® Omega, BMG Labtech, Ortenberg, Germany). Percentage viability was normalised to a plate-wise, time-matched 0 mM control using the equation: , where  is the absorbance of the test sample,  is the absorbance of the control sample and  is the absorbance of the blank well with fibronectin only. Three biological repeats were performed.

### 2.7 Statistical Analysis

Statistical analysis and graphics were performed using GraphPad Prism (version 10.2.1, GraphPad Software, San Diego, CA, USA). Flow cytometry means FI data were analysed using multiple unpaired *t*-tests with Welch’s correction. ROS MFI data were analysed using Kruskal-Wallis tests with post-hoc Dunn’s test for multiple comparisons. Viability data were analysed using Welch’s ANOVA with post-hoc Dunnett’s T3 multiple comparison test. Dark toxicity was investigated in the SL group using nonlinear regression, which was performed using a four-parameter logistic model (log[inhibitor] vs. response—variable slope), constraining the top plateau to 100%. Nonlinear regression did not converge to a sigmoidal fit and no reliable IC_50_ values could be determined. Therefore, statistical differences between each concentration and the 0 mM control were assessed using Welch’s ANOVA with post-hoc Dunnett’s T3 multiple comparison test. Statistical significance for all tests was considered at *p-*values < 0.05.

## 3 Results

### 3.1 Cellular Uptake of 5-ALA

**Figure 2:**
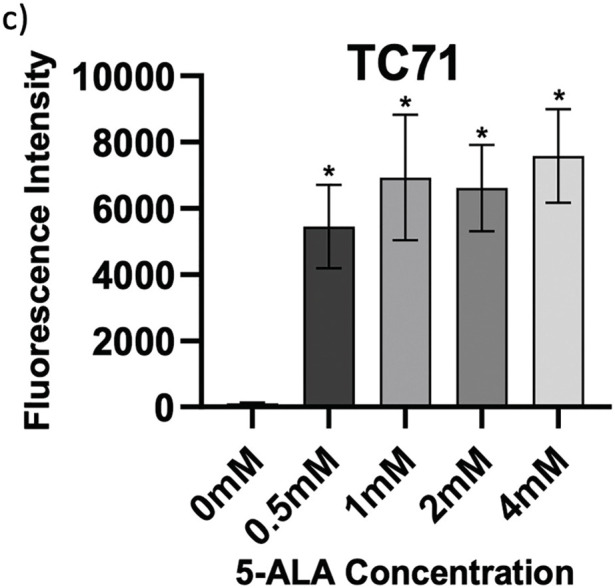

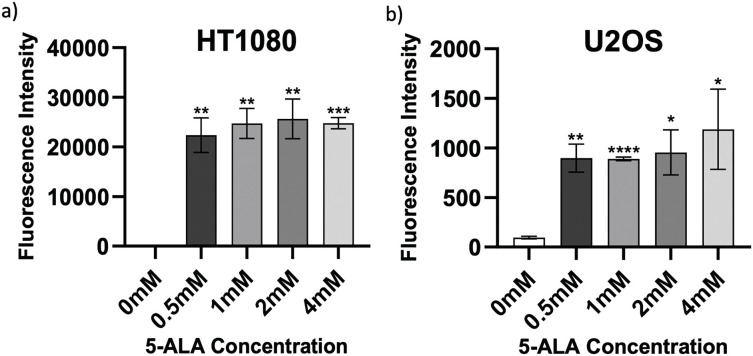
Intragroup comparison of uptake at all concentrations of 5-ALA across the panel of cell lines. Bar charts showing mean FI and significant differences in mean FI at all concentrations of 5-ALA compared to the 0 mM control for the panel of cell lines. (**a**) HT1080, (**b**) U2OS, (**c**) TC71. The *x*-axis represents the various 5-ALA concentrations, the *y*-axis represents mean FI (linear scale, note scales are not the same for a-c) caused by detection of PpIX. Asterisks denote significant differences between the mean FI of all treatment concentrations and the 0 mM control group, analysed using multiple unpaired *t*-tests with Welch’s correction. *p < 0.05; **p < 0.01; ***p < 0.001; ****p < 0.0001. Error bars show mean FI ± SD, 10,000 events were recorded during all repeats (n = 3)

Determining accumulation of PpIX, therefore 5-ALA, in bone sarcoma cell lines was the imperative initial step, before attributing an increase in ROS production or reduction in cell viability to 5-ALA-PDT. Following incubation with 5-ALA, accumulation of PpIX was detected via flow cytometry in all cell lines (Fig. 1a–c). Significantly higher levels of PpIX MFI were shown in all cell lines at all treatment concentrations (0.5, 1, 2 and 4 mM) of 5-ALA compared to the 0 mM control (Fig. 2a–c) (*p* < 0.05) after three biological repeats.

### 3.2 ROS Production by 5-ALA-PDT

**Figure 3:**
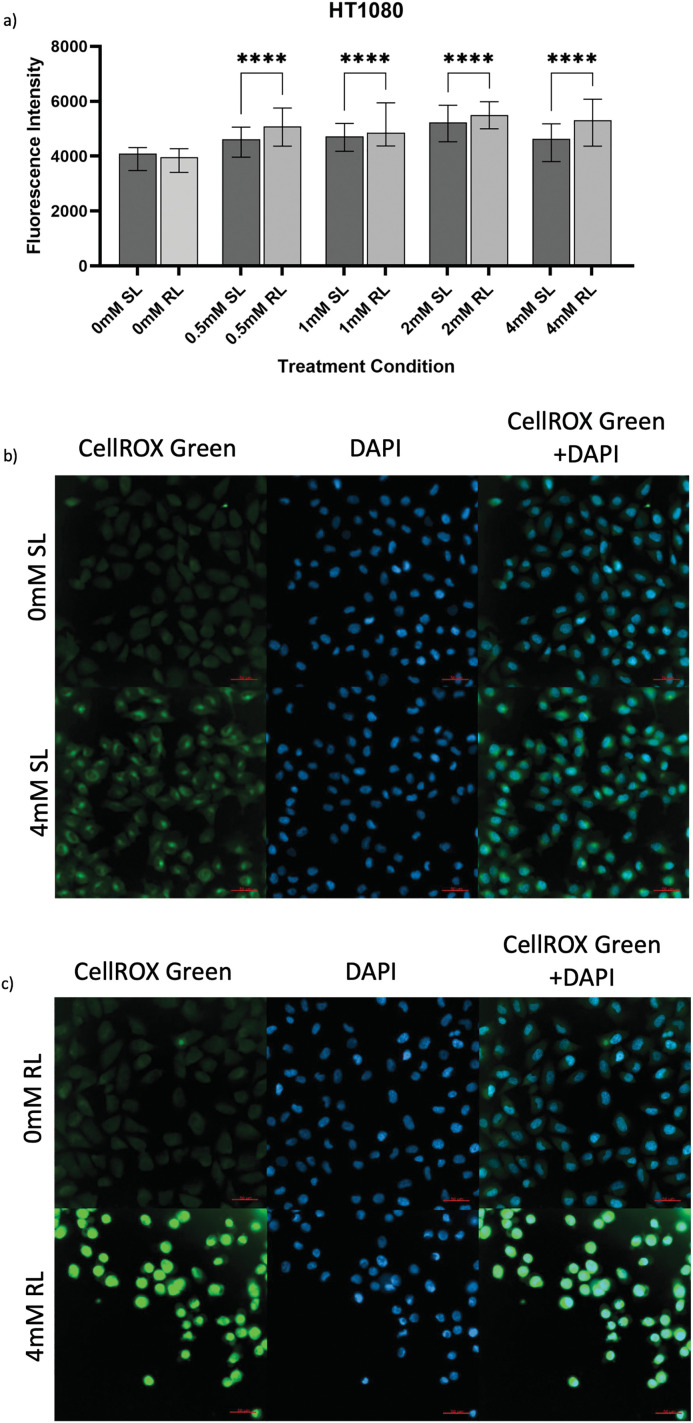
Trends in CellROX Green MFI and relative ROS levels across all 5-ALA concentrations in HT1080 cells treated with sham-red and red light. (**a**) Bar graph showing the MFI of HT1080 cells from each treatment condition and significant differences between the same concentration of SL and RL-cells. The *x*-axis represents the various treatment conditions, the *y*-axis represents fluorescence intensity (linear scale). Error bars show MFI ± IQR. Asterisks denote concentrations at which RL-cells had significantly higher MFIs than the same concentration of SL-cells, following statistical analysis with Kruskal-Wallis with post-hoc Dunn’s multiple comparison test. No asterisk or bracket means *p* ≥ 0.05; ****p < 0.0001. (**b**) Fluorescence microscopy images of HT1080 cells treated with 0 or 4 mM 5-ALA and SL or (**c**) RL, and stained with CellROX Green and DAPI nuclear stain. Images show CellROX Green fluorescence only (left), DAPI only (middle) and CellROX Green and DAPI fluorescence (right) for 0 mM 5-ALA (top) and 4 mM (bottom) 5-ALA. Scale bar (red, bottom right-hand corner) represents 50 μm

**Figure 4:**
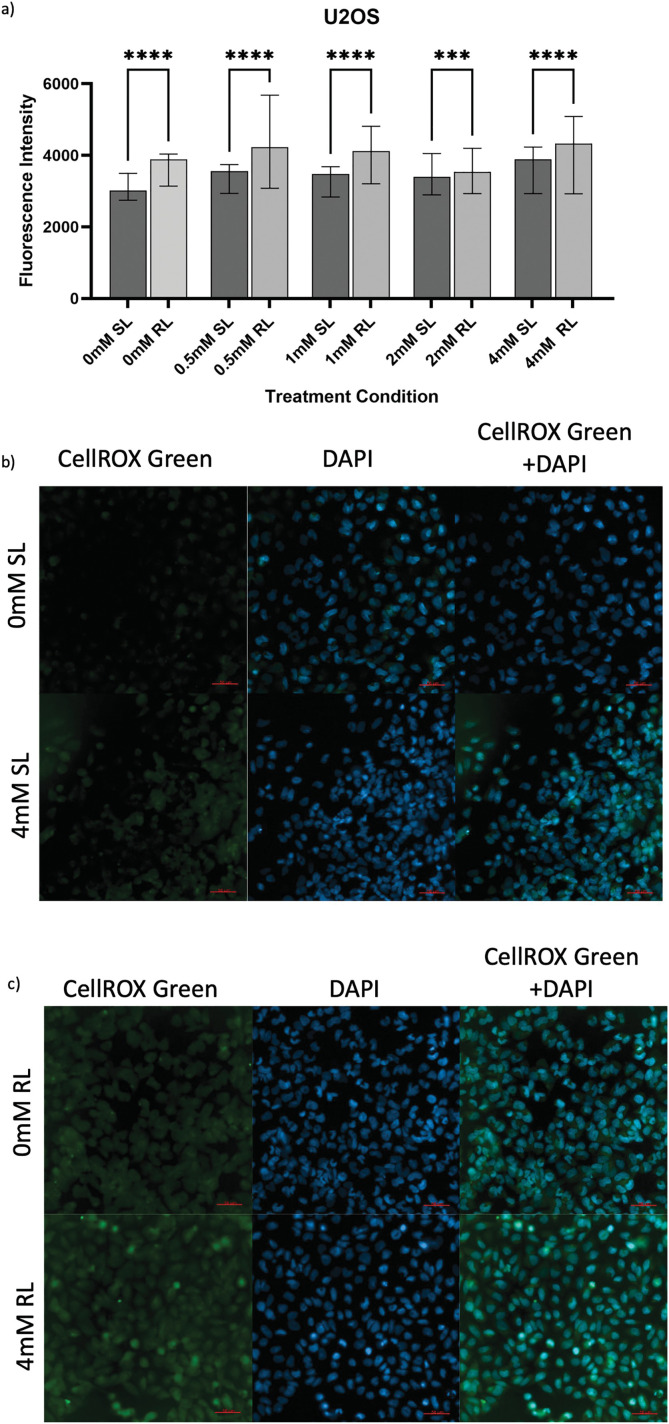
Trends in CellROX Green MFI and relative ROS levels across all 5-ALA concentrations in U2OS cells treated with sham-red and red light. (**a**) Bar graph showing the MFI of U2OS cells from each treatment condition and significant differences between the same concentration of SL and RL-cells. The *x*-axis represents the various treatment conditions, the *y*-axis represents fluorescence intensity (linear scale). Error bars show MFI ± IQR. Asterisks denote concentrations at which RL-cells had significantly higher MFIs than the same concentration of SL-cells, following statistical analysis with Kruskal-Wallis with post-hoc Dunn’s multiple comparison test. ***p < 0.001; ****p < 0.0001. (**b**) Fluorescence microscopy images of HT1080 cells treated with 0 or 4 mM 5-ALA and SL or (**c**) RL, and stained with CellROX Green and DAPI nuclear stain. Images show CellROX Green fluorescence only (left), DAPI only (middle) and CellROX Green and DAPI fluorescence (right) for 0 mM 5-ALA (top) and 4 mM (bottom) 5-ALA. Scale bar (red, bottom right-hand corner) represents 50 μm

**Figure 5:**
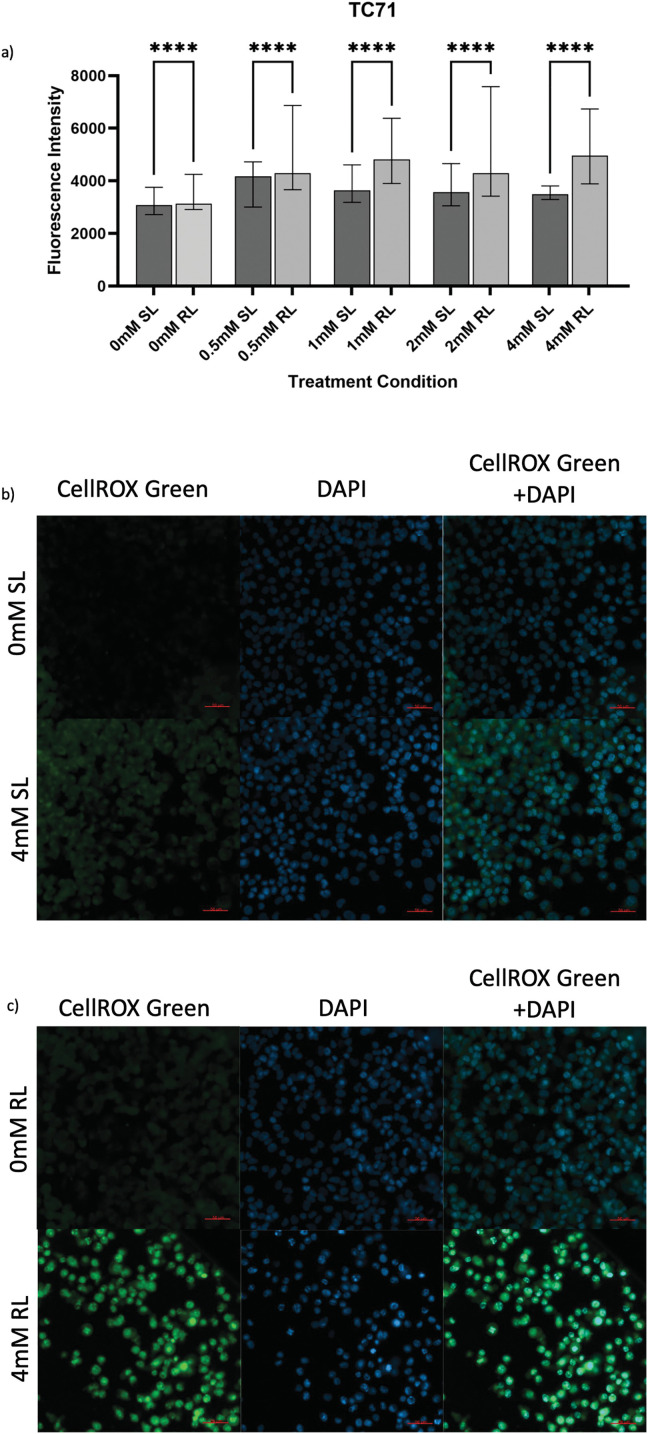
Trends in CellROX Green MFI and relative ROS levels across all 5-ALA concentrations in TC71 cells treated with sham-red and red light. (**a**) Bar graph showing the MFI of TC71 cells from each treatment condition and significant differences between the same concentration of SL and RL-cells. The *x*-axis represents the various treatment conditions, the *y*-axis represents fluorescence intensity (linear scale). Error bars show MFI ± IQR. Asterisks denote concentrations at which RL-cells had significantly higher MFIs than the same concentration of SL-cells, following statistical analysis with Kruskal-Wallis with post-hoc Dunn’s multiple comparison test. ****p < 0.0001. (**b**) Fluorescence microscopy images of HT1080 cells treated with 0 or 4 mM 5-ALA and SL or (**c**) RL; and stained with CellROX Green and DAPI nuclear stain. Images show CellROX Green fluorescence only (left), DAPI only (middle) and CellROX Green and DAPI fluorescence (right) for 0 mM 5-ALA (top) and 4 mM (bottom) 5-ALA. Scale bar (red, bottom right-hand corner) represents 50 μm

To assess levels of ROS production induced by 5-ALA-PDT, cells were stained with CellROX Green before imaging and analysis. Figs. 3–5 show fluorescence microscopy images and pairwise comparisons for SL-treated cells (SL-cells) and RL-treated cells (RL-cells) in each cell line. As expected, ROS were detected in all samples, due to production during normal cellular activity [61]. However, all cell lines also demonstrated significantly higher CellROX MFI in samples following treatment with PDT (treatment with 5-ALA and RL), rather than SL or 5-ALA alone, suggesting increased ROS production following 5-ALA-PDT.

#### 3.2.1 HT1080

The success of 5-ALA-PDT in increasing ROS production was seen by the significantly higher MFIs of all treatment concentrations of RL-cells in comparison to their SL counterparts (Fig. 3a–c) (*p* < 0.0001). All treatment concentrations of RL and SL-cells had significantly higher fluorescence than 0 mM SL and RL-cells (*p* < 0.0001). The MFI of 0 mM SL and RL-cells yielded an insignificant difference. SL-cells treated with 2 mM demonstrated the greatest increase in ROS production, having a significantly higher MFI than 0, 0.5, 1 and 4 mM (*p* < 0.0001). Again, 2 mM appeared to induce significantly more ROS production than all other 5-ALA concentrations in RL-cells (*p* < 0.0001).

#### 3.2.2 U2OS

In U2OS, the overall increased production of ROS following 5-ALA-PDT was suggested by all concentrations of RL-cells having significantly higher fluorescence than their SL counterparts (Fig. 4a–c), (*p* < 0.0001 for 0, 0.5, 1 and 4 mM; *p* = 0.0004 for 2 mM). 0 mM RL-cells had a significantly higher MFI than 0, 0.5, 1 and 2 mM SL-cells and 2 mM RL-cells (*p* < 0.0001). All treatment concentrations of RL and SL-cells had higher fluorescence than 0 mM SL-cells (*p* < 0.0001). 0.5, 1 and 4 mM were more efficacious in increasing ROS production in RL-cells than 2 mM (*p* < 0.0001).

#### 3.2.3 TC71

5-ALA-PDT increased ROS production in TC71 cells. All treatment concentrations of RL-cells were significantly more fluorescent than 0 mM SL and RL-cells and all concentrations of SL-cells (Fig. 5a–c) (*p* < 0.0001). 0 mM RL-cells had a significantly higher MFI than 0 mM SL-cells (*p* < 0.0001), suggesting RL-induced ROS production. All treatment concentrations of SL-cells had higher MFIs than 0 mM SL-cells and all, apart from 4 mM SL-cells, had higher MFIs than 0 mM RL-cells (*p* < 0.0001). In RL-cells, 0.5, 1 and 4 mM were more effective in enhancing ROS production than 2 mM (*p* = 0.012, *p* < 0.0001 and *p* < 0.0001, respectively).

### 3.3 Cytotoxic Effects of 5-ALA-PDT

#### 3.3.1 Raw Absorbance Data

**Figure 6:**
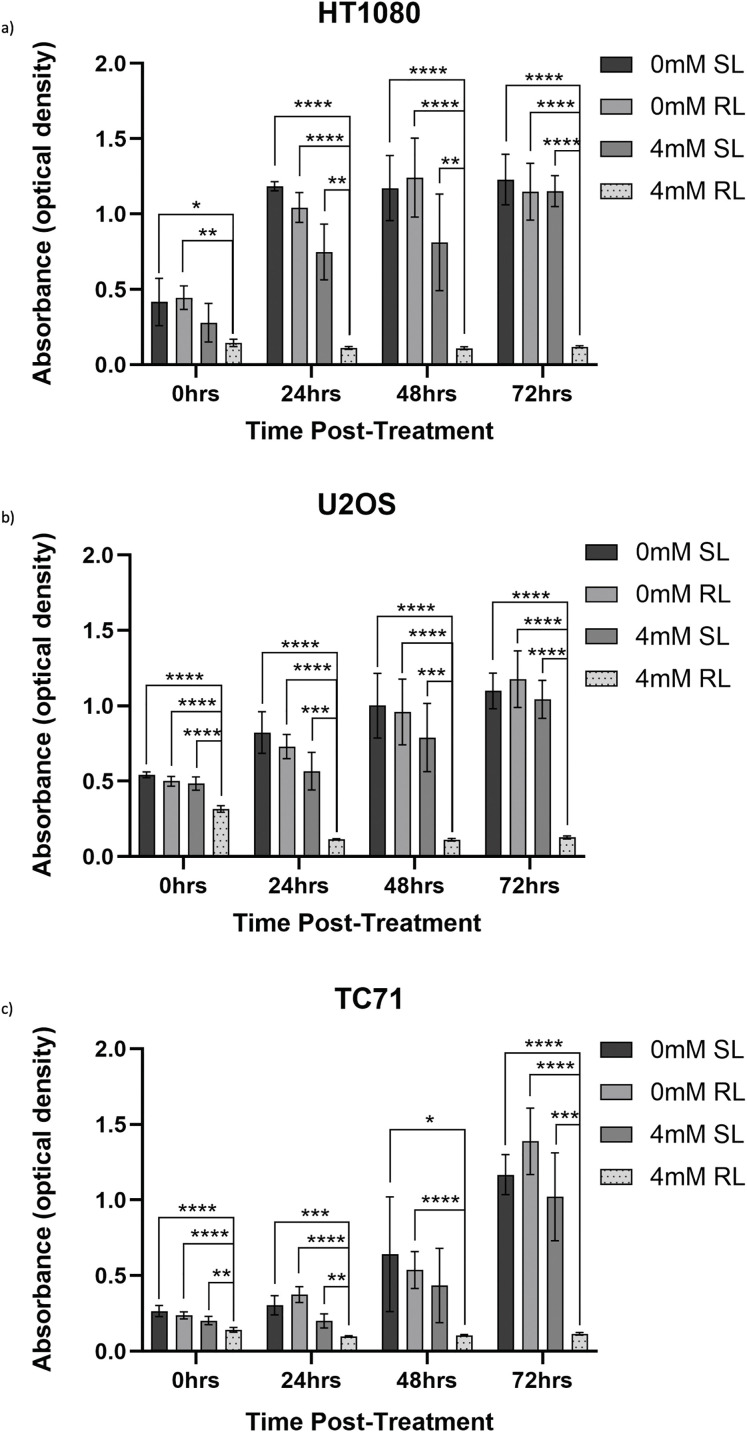
Trends in raw absorbance data 0–72 h post-treatment in sham-red and red light-treated cells at 0 and 4 mM 5-ALA. Bar graphs showing mean raw absorbance values, measured as optical density, and significant differences between 4 mM RL and 0 mM SL, 0 mM RL and 4 mM SL following CCK-8 assays. The *x*-axis represents the various time points, the *y*-axis represents absorbance values (linear scale). Asterisks denote time points at which 4 mM RL had a significantly lower absorbance value than the other three plotted treatment conditions and the level of this significance, following analysis with Welch’s ANOVA and post-hoc Dunnett’s T3 multiple comparison test. No asterisk or bracket means *p* ≥ 0.05; *p < 0.05; **p < 0.01; ***p < 0.001; ****p < 0.0001. Error bars show mean ± SD, n = 9 for each bar. (**a**) HT1080, (**b**) U2OS, (**c**) TC71

**Figure A1:**
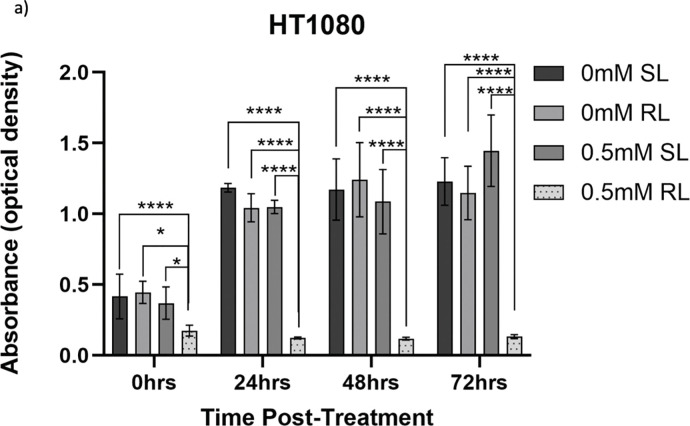

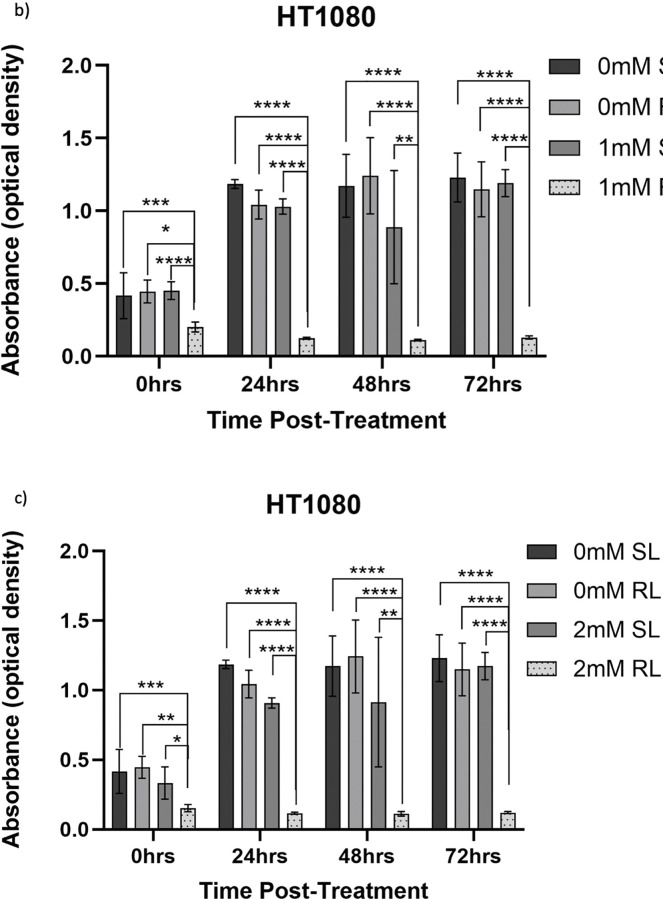
Trends in raw absorbance data 0–72 h post-treatment in sham-red and red light-treated HT1080 cells at 0 mM–2 mM 5-ALA. Bar graphs showing mean raw absorbance values, measured as optical density, for HT1080 cells. The *x*-axis represents the various time points, the *y*-axis represents absorbance values (linear scale). Error bars show mean ± SD, n = 9 for each bar. (**a**) Denotes significant differences between 0.5 mM RL and 0 mM SL, 0 mM RL and 0.5 mM SL following CCK-8 assays. (**b**) Denotes significant differences between 1 mM RL and 0 mM SL, 0 mM RL and 1 mM SL following CCK-8 assays. (**c**) Denotes significant differences between 2 mM RL and 0 mM SL, 0 mM RL and 2 mM SL following CCK-8 assays. Analysis was performed with Welch’s ANOVA and post-hoc Dunnett’s T3 multiple comparison test. *p < 0.05; **p < 0.01; ***p < 0.001; ****p < 0.0001

**Figure A2:**
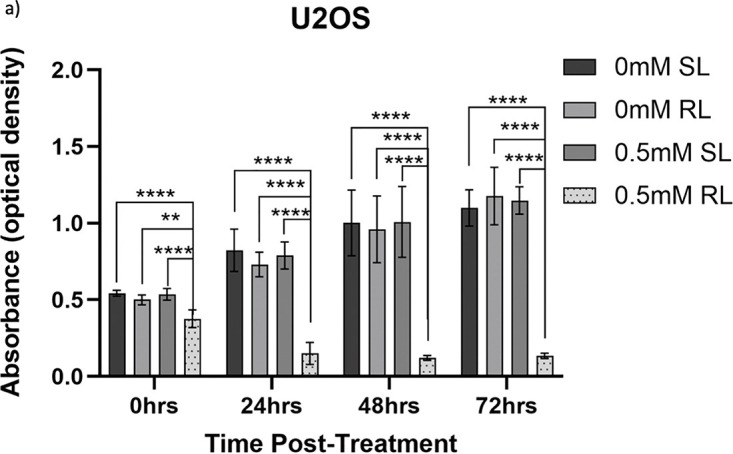

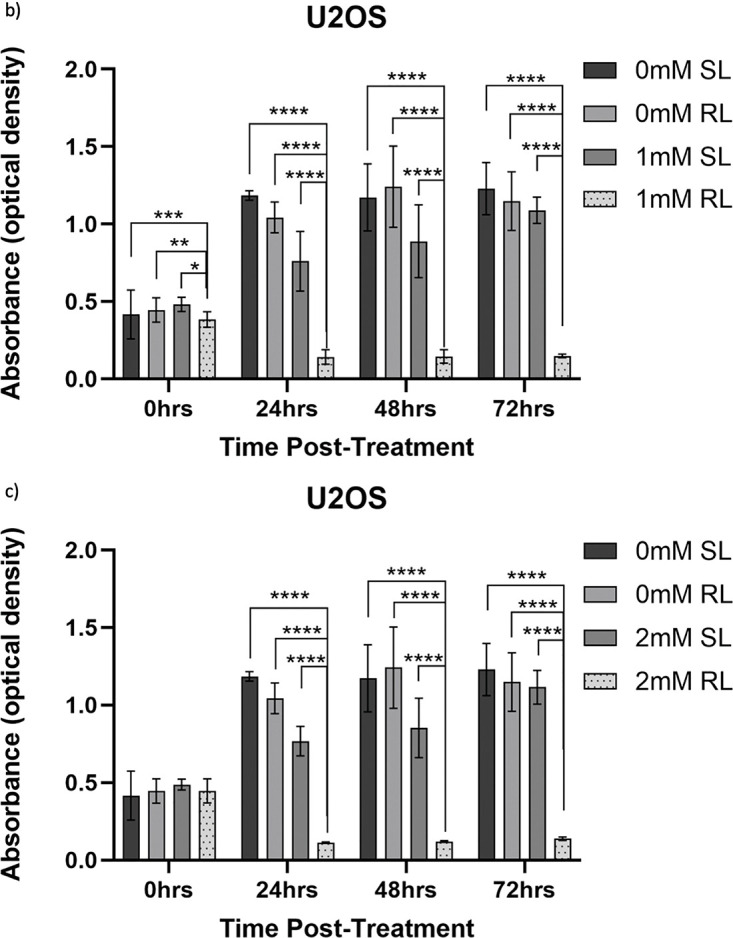
Trends in raw absorbance data 0–72 h post-treatment in sham-red and red light-treated U2OS cells at 0 mM–2 mM 5-ALA. Bar graphs showing mean raw absorbance values, measured as optical density, for U2OS cells. The *x*-axis represents the various time points, the *y*-axis represents absorbance values (linear scale). Error bars show mean ± SD, n = 9 for each bar. (**a**) Denotes significant differences between 0.5 mM RL and 0 mM SL, 0 mM RL and 0.5 mM SL following CCK-8 assays. (**b**) Denotes significant differences between 1 mM RL and 0 mM SL, 0 mM RL and 1 mM SL following CCK-8 assays. (**c**) Denotes significant differences between 2 mM RL and 0 mM SL, 0 mM RL and 2 mM SL following CCK-8 assays. Analysis was performed with Welch’s ANOVA and post-hoc Dunnett’s T3 multiple comparison test. No asterisk or bracket means *p* ≥ 0.05; *p < 0.05; **p < 0.01; ***p < 0.001; ****p < 0.0001

**Figure A3:**
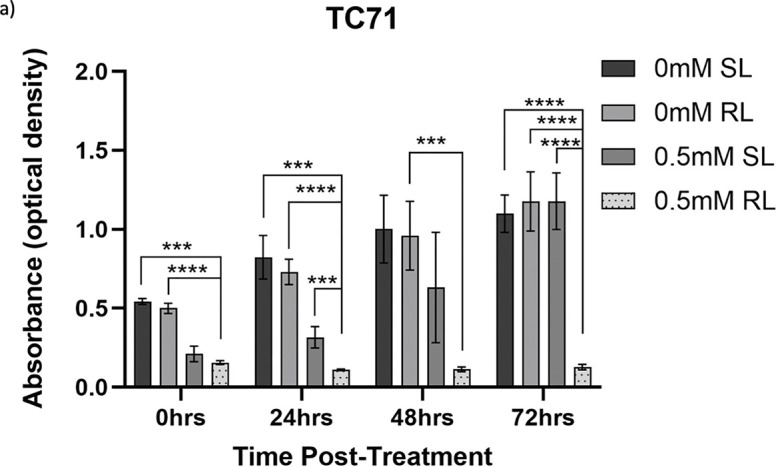

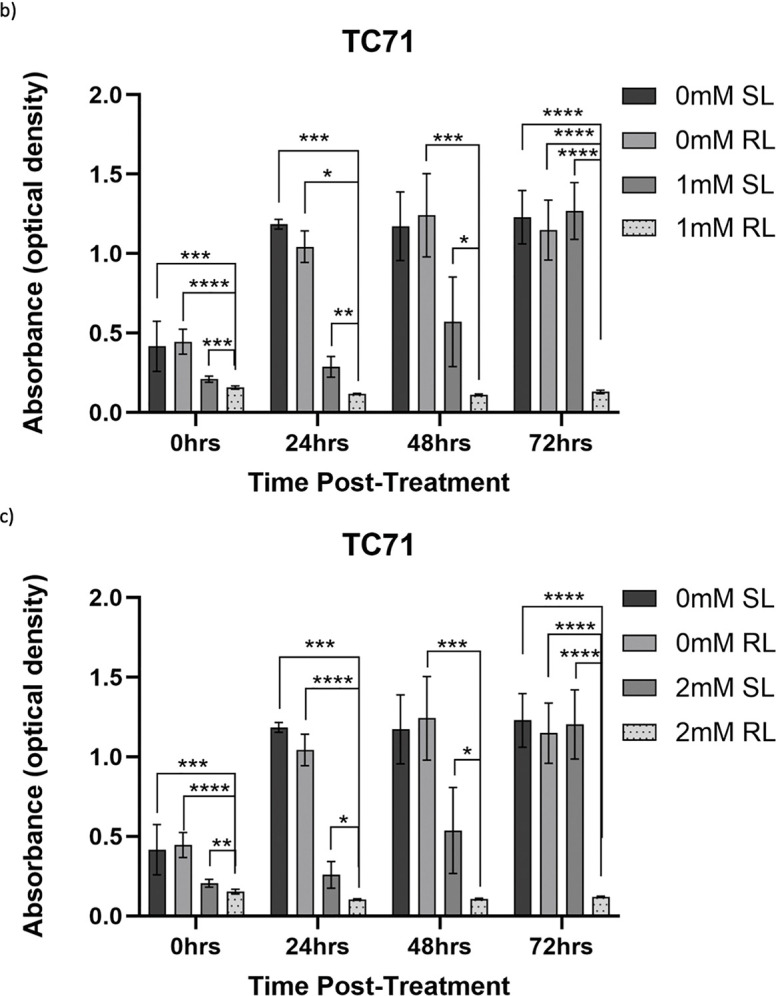
Trends in raw absorbance data 0–72 h post-treatment in sham-red and red light-treated TC71 cells at 0 mM–2 mM 5-ALA. Bar graphs showing mean raw absorbance values, measured as optical density, for TC71 cells. The *x*-axis represents the various time points, the *y*-axis represents absorbance values (linear scale). Error bars show mean ± SD, n = 9 for each bar. (**a**) Denotes significant differences between 0.5 mM RL and 0 mM SL, 0 mM RL and 0.5 mM SL following CCK-8 assays. (**b**) Denotes significant differences between 1 mM RL and 0 mM SL, 0 mM RL and 1 mM SL following CCK-8 assays. (**c**) Denotes significant differences between 2 mM RL and 0 mM SL, 0 mM RL and 2 mM SL following CCK-8 assays. Analysis was performed with Welch’s ANOVA and post-hoc Dunnett’s T3 multiple comparison test. No asterisk or bracket means *p* ≥ 0.05; *p < 0.05; **p < 0.01; ***p < 0.001; ****p < 0.0001

Raw absorbance data, measured as optical density, obtained from CCK-8 assays are presented to provide the unprocessed measurements underlying the normalised viability results, which have been analysed in Section 3.3.2. Fig. 6a–c exhibits CCK-8 absorbance data, measured as optical density, showing values for 0 and 4 mM, RL and SL-cells across all time points. Figs. A1–A3 exhibit raw absorbance data for all other concentrations for each cell line. At 72 h, the only significant result for all cell lines (from the specific data analysed) was 0.5, 1, 2 and 4 mM RL-cells having significantly lower absorbance values than the same concentration of SL-cells, 0 mM SL and 0 mM RL (*p* < 0.001).

**Figure 7:**
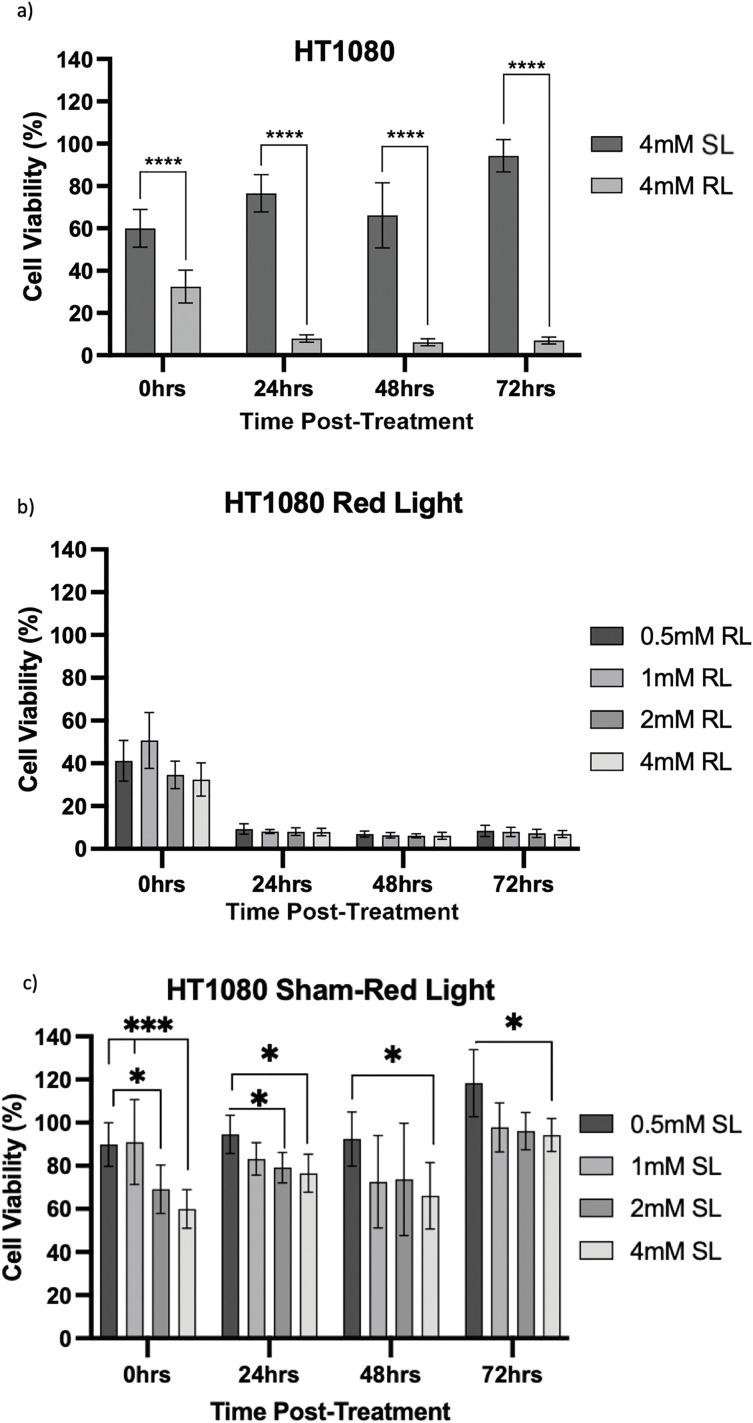
Trends in HT1080 cell viability 0–72 h post-treatment in sham-red and red light-treated cells at 0.5, 1, 2 and 4 mM 5-ALA. Bar graphs showing mean cell viability (%) calculated following CCK-8 assays. The *x*-axis represents the various time points, the *y*-axis represents percentage cell viability (linear scale). Error bars show mean ± SD, n = 9 for each bar. (**a**) Trends in viability of 4 mM SL and RL-cells. Asterisks denote time points at which 4 mM RL-cells had significantly lower viabilities than 4 mM SL-cells. (**b**) Trends in viability of RL-cells. Asterisks denote time points at which there was a significant difference in viabilities of RL-cells treated with different concentrations of 5-ALA. (**c**) Trends in viability of SL-cells. Asterisks denote time points at which there was a significant difference in viabilities of SL-cells treated with different concentrations of 5-ALA. Statistical analysis was performed using Welch’s ANOVA and post-hoc Dunnett’s T3 multiple comparison test. No asterisk or bracket means *p* ≥ 0.05; *p < 0.05; ***p < 0.001; ****p < 0.0001

**Figure 8:**
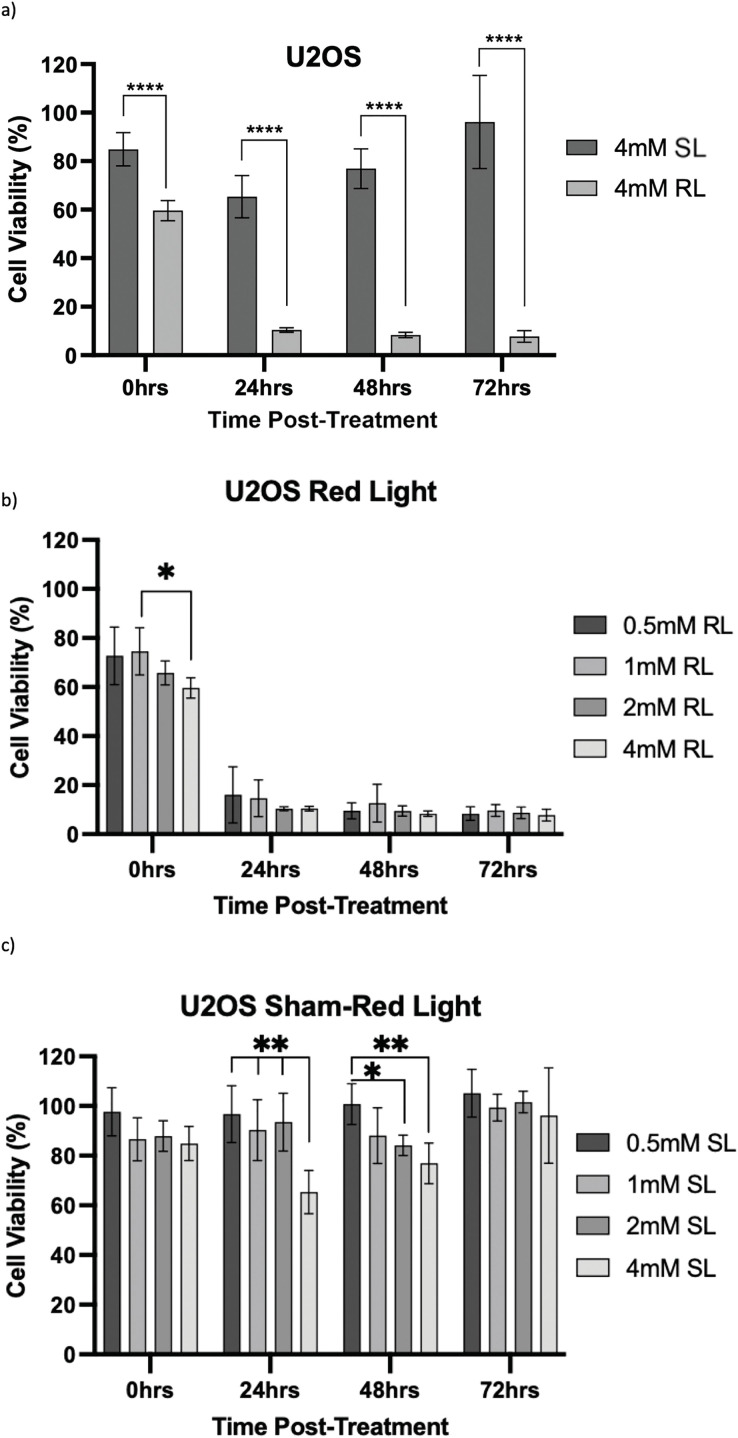
Trends in U2OS cell viability 0–72 h post-treatment in sham-red and red light-treated cells at 0.5, 1, 2 and 4 mM 5-ALA. Bar graphs showing mean cell viability (%) calculated following CCK-8 assays. The *x*-axis represents the various time points, the *y*-axis represents percentage cell viability (linear scale). Error bars show mean ± SD, n = 9 for each bar. (**a**) Trends in viability of 4 mM SL and RL-cells. Asterisks denote time points at which 4 mM RL-cells had significantly lower viabilities than 4 mM SL-cells. (**b**) Trends in viability of RL-cells. Asterisks denote time points at which there was a significant difference in viabilities of RL-cells treated with different concentrations of 5-ALA. (**c**) Trends in viability of SL-cells. Asterisks denote time points at which there was a significant difference in viabilities of SL-cells treated with different concentrations of 5-ALA. Statistical analysis was performed using Welch’s ANOVA and post-hoc Dunnett’s T3 multiple comparison test. No asterisk or bracket means *p* ≥ 0.05; *p < 0.05; **p < 0.01; ****p < 0.0001

**Figure 9:**
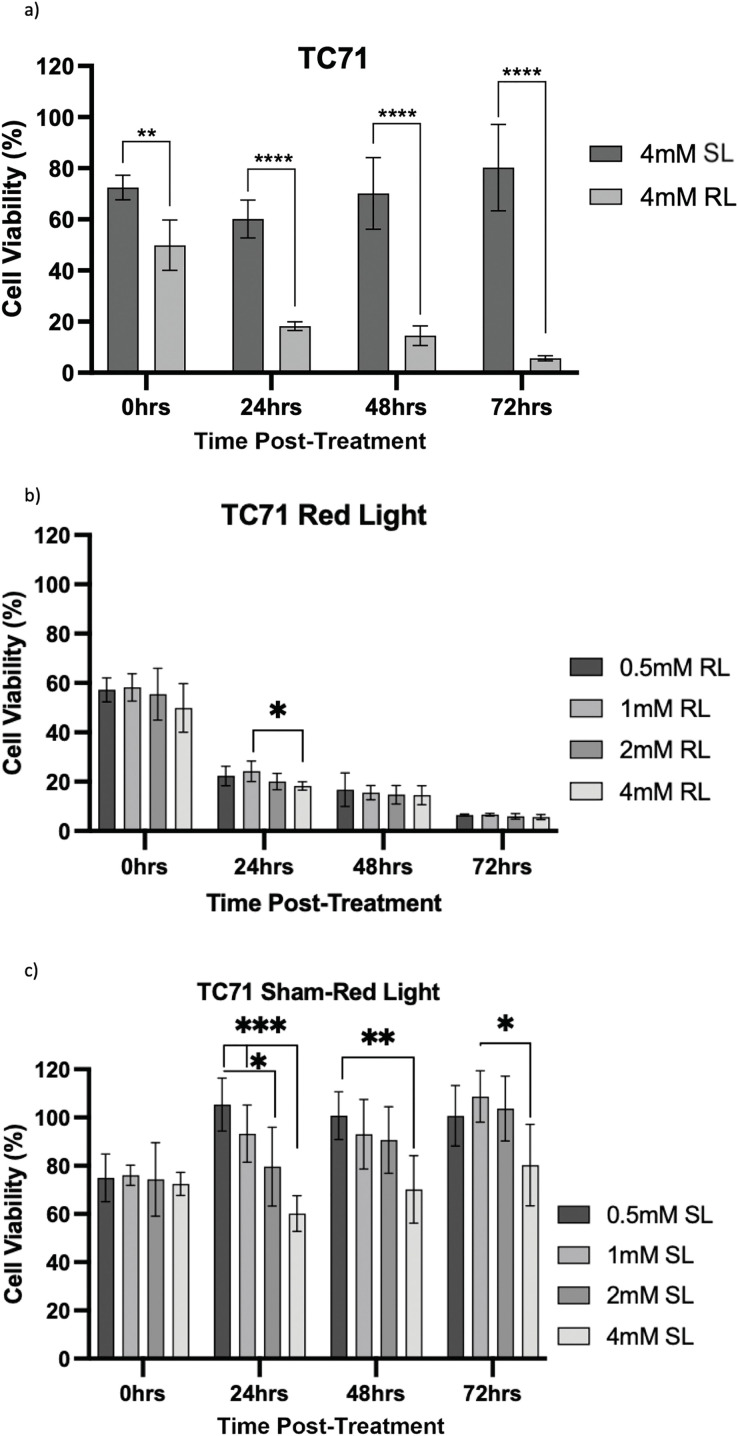
Trends in TC71 cell viability 0–72 h post-treatment in sham-red and red light treated cells at 0.5, 1, 2 and 4 mM 5-ALA. Bar graphs showing mean cell viability (%) calculated following CCK-8 assays. The *x*-axis represents the various time points, the *y*-axis represents percentage cell viability (linear scale). Error bars show mean ± SD, n = 9 for each bar. (**a**) Trends in viability of 4 mM SL and RL-cells. Asterisks denote time points at which 4 mM RL-cells had significantly lower viabilities than 4 mM SL-cells. (**b**) Trends in viability of RL-cells. Asterisks denote time points at which there was a significant difference in viabilities of RL-cells treated with different concentrations of 5-ALA. (**c**) Trends in viability of SL-cells. Asterisks denote time points at which there was a significant difference in viabilities of SL-cells treated with different concentrations of 5-ALA. Statistical analysis was performed using Welch’s ANOVA and post-hoc Dunnett’s T3 multiple comparison test. No asterisk or bracket means *p* ≥ 0.05; *p < 0.05; **p < 0.01; ***p < 0.001; ****p < 0.0001

Viability was then calculated by normalising optical density values against the plate-wise time-matched 0 mM control and is shown for HT1080 (Fig. 7a–c), U2OS (Fig. 8a–c) and TC71 (Fig. 9a–c).

#### 3.3.2 Viability Data

HT1080, U2OS and TC71 cells were incubated for 4 h with increasing concentrations of 5-ALA and subsequently treated with SL or RL. After 0, 24, 48 and 72 h, the viability of cells was assessed. Analyses from three biological repeats are summarised for each cell line in Figs. 7–9. Viability at 0 mM cannot be calculated due to its presence as a constant in the viability equation. At all time points, RL-cells were significantly less viable than SL-cells. By 24 h, all concentrations of RL-cells had significantly lower viabilities than all concentrations of SL-cells in every cell line (*p* < 0.0001). There were no significant differences present between the various 5-ALA concentrations in RL-cells by 72 h. However, the viability of 4 mM SL cells was significantly lower than 0.5 mM SL-cells in HT1080s (*p* = 0.029) and 1 mM SL-cells in TC71s (*p* = 0.021).

HT1080

At 0 h, all concentrations of RL-cells, apart from 1 mM RL-cells, had significantly lower viabilities than all concentrations of SL-cells. At 24, 48 and 72 h, all concentrations of RL-cells were less viable than all concentrations of SL-cells (*p* < 0.01). This is displayed for 4 mM concentration by Fig. 7a. There was no dose-dependent effect across 5-ALA concentrations at any time point in RL-cells (Fig. 7b). A stable IC_50_ could not be reliably estimated for the SL condition because the response was shallow. At 0 h, 4 mM SL-cells were less viable than 1 and 0.5 mM SL-cells, and continued to be less viable than 0.5 mM SL-cells at all other time points (*p* < 0.05). At 0 and 24 h 2 mM SL-cells were less viable than 0.5 mM SL-cells points (*p* < 0.05) (Fig. 7c). By 72 h, the range in viability was 5.1%–11.9% and 80.1%–127.6% for RL and SL-cells, respectively, across treatment concentrations of 5-ALA.

U2OS

The viability of all concentrations of RL-cells was significantly lower than all concentrations of SL-cells at 24, 48 and 72 h (*p* < 0.01). This is displayed for the 4 mM concentration by Fig. 8a. At 0 h, only 2 and 4 mM RL-cells were significantly less viable than their SL counterparts. Additionally, at 0 h, 4 mM RL-cells had lower viability than 1 mM RL-cells, with mean viabilities of 59.6% and 74.5%, respectively, suggesting greater efficacy of higher 5-ALA concentrations (*p* < 0.05) (Fig. 8b). However, beyond 0 h, no significant differences in the viabilities of RL-cells between treatment concentrations were detected. RL-cells also showed no further significant decrease in viability at any concentration beyond 48 h, however, SL-cells continued to show increased viability up to 72 h (*p* < 0.05). A stable IC_50_ could not be reliably determined due to the shallow nature of the response. However, there was evidence of dark toxicity at higher 5-ALA concentrations, with 4 mM SL-cells having significantly lower viability than 0.5, 1 and 2 mM SL-cells at 24 h and 0.5 mM at 48 h (*p* < 0.01) (Fig. 8c). 2 mM SL-cells were also significantly less viable than 0.5 mM SL-cells at 48 h (*p* < 0.01) (Fig. 8c). However, by 72 h, there were no significant differences between the viabilities of SL-cells. By 72 h, the range in viability was 5.3%–13.6% and 73.6%–128.0% for RL and SL-cells, respectively, across all treatment concentrations of 5-ALA.

TC71

At 0 h, all concentrations of RL-cells were significantly less viable than 0.5, 1 and 4 mM SL-cells (*p* < 0.05). This is displayed for the 4 mM concentration by Fig. 9a. At 24, 48 and 72 h, all concentrations of RL-cells were significantly less viable than all concentrations of SL-cells (*p* < 0.0001). At 24 h, 4 mM RL-cells were significantly less viable than 1 mM RL-cells (*p* = 0.042), with mean viabilities of 18.2% and 25.2%, respectively (Fig. 9b). However, this was the only time point at which a difference was present between the efficacy of 5-ALA concentrations in RL-cells. A stable IC_50_ could not be reliably estimated for the dark condition because the response was shallow. In SL-cells, 4 mM cells were significantly less viable than 0.5 and 1 mM cells at 24 h (*p* < 0.0001). 4 mM SL continued to appear more toxic, having a significantly lower viability than 0.5 mM SL-cells (*p* = 0.0026), then 1 mM SL-cells (*p* = 0.021), at 48 and 72 h, respectively (Fig. 9c). 2 mM SL-cells were also significantly less viable than 0.5 mM SL-cells at 24 h (*p* = 0.036) (Fig. 9c). RL-cells showed a significant decrease and SL-cells a significant increase, in viability between adjacent time points up to 72 h, suggesting continued trends (*p* < 0.05). By 72 h, the range in viability was 4.4%–7.7% and 61.5%–118.7% for RL and SL-cells, respectively, across all treatment concentrations of 5-ALA.

## 4 Discussion

A major limitation in sarcoma management is the frequency of and poor outcomes associated with positive surgical margins. Attempting to limit morbidity through less radical surgical resections, rather than amputation, can result in inadvertent positive margins, which are associated with a significantly increased risk of LR and poorer prognoses [13,61]. A treatment approach combining resection with FGS and intraoperative PDT may optimise surgical management for sarcoma, minimising healthy tissue loss and maintaining post-operative limb function, whilst simultaneously reducing LR rates. The main findings of this study were increased uptake of 5-ALA by bone sarcoma cells and significantly increased ROS production and reduced cellular viability, following treatment with 5-ALA and RL, demonstrating the potential for future application of 5-ALA-PDT as an adjuvant therapy for bone sarcoma.

Several *in vitro* and *in vivo* studies demonstrate 4 h as the optimal drug-light interval to enable successful 5-ALA uptake and conversion to PpIX, prior to irradiation [30,62,63]. The results from this study support this, with all concentrations of 5-ALA showing significant uptake and conversion to PpIX in all cell lines, after incubation for 4 h. This supports current guidance regarding oral delivery of 5-ALA for FGS in neurosurgery, given 2–4 h prior to surgery. If used in sarcoma for both FGS and PDT, intravenous or topical delivery will likely be required due to the procedural length of large resections and the need for re-optimisation of intracellular 5-ALA concentration. Optimisation of intracellular concentrations was not in the realm of this study; however, the lack of significant differences in levels of uptake between 0.5–4 mM meant uptake was not seen in a dose-dependent manner at these concentrations and may suggest cells were saturated with 5-ALA, highlighting the need for further dose-titration investigations.

The absence of control cell lines must be noted, meaning reduced 5-ALA uptake, ROS production and increased cell viability were not shown in a non-malignant control. Future studies should consider the use of human fibroblasts or primary osteoblasts as a means of control. However, using monolayer cells, which lack normal tumour physiology, means this may be difficult in an *in vitro* setting. Despite this, 5-ALA has been shown to experience selective uptake by malignant cells and to be non-toxic to non-malignant control cell lines in comparison [63,64]. In the context of FGS, 5-ALA is well established and selectively taken up by malignant cells, but this is without the more penetrable wavelengths and proximity of excitation light sources required to stimulate PDT. There is potential to develop preclinical spheroid models, more closely reflecting the depth of light penetrance required for successful intraoperative PDT, than what is required for success in monolayers. Multicellular spheroids, containing sarcoma cells and non-malignant cells, could be developed to monitor the effects of 5-ALA-PDT in healthy tissue adjacent to tumours, before progression into *in vivo* murine models.

In the UK, 5-ALA-PDT is used for skin malignancies, however, the topical application used in this setting means distribution can be greater controlled compared to oral or intravenous delivery, where the reliance would be on selective uptake by cancer cells and clearance from healthy tissues. Intraoperative application of 5-ALA may be performed topically to the wound bed, with the light source only directed towards the area of interest. Therefore, activation of 5-ALA-PDT would be spatially confined, increasing specificity and limiting effects on surrounding non-malignant cells. Once the best method of delivery is established, 5-ALA uptake requires more attention in terms of malignant selectivity and intracellular concentrations, before *in vivo* use.

Significantly increased ROS production was experienced in cells treated with 5-ALA-PDT compared to SL-cells. This mirrors findings of studies using CellROX Green [65] and alternative cellular ROS assays [63] to detect ROS following 5-ALA-PDT. However, our study is one of the few to investigate this. Several papers assess 5-ALA uptake and cell viability only [39,54,59], without providing evidence of ROS production before concluding successful PDT has occurred. ROS production caused by 5-ALA is independent of PDT (dark toxicity) and comparisons between production in SL and RL-cells at various concentrations have not been shown by other studies. CellROX Green detects superoxide but is not sensitive to hydrogen peroxide [66], therefore, may not have detected all ROS present. This study did not establish the types of ROS produced or the extent of damage caused, so it cannot suggest prevailing mechanisms of cell death subsequent to PDT in bone sarcoma. Future experimentation could make use of complementary probes to detect and characterise the location and quantify the amount of singlet oxygen and superoxide [67,68]. The increase in ROS production was not observed in a consistent dose-dependent manner, with different concentrations being most effective in different cell lines.

RL counterparts produced significantly higher fluorescence than SL-cells for all concentrations of 5-ALA in U2OS and TC71 cell lines. This means that RL was causing an increase in ROS, therefore causing potential toxicity, independent of 5-ALA in the 0 mM groups. It has been suggested that delivery of RL at dosages above 15 J/cm^2^ can damage mitochondria and cause excessive oxidative stress [69,70]. This study used a fluence of 20 J/cm^2^; based on significant results seen in terms of both ROS production and reduction in cell viability, future work should consider delivering light at lower, variable doses and monitoring the impact, if any, this has. Changing radiation parameters may minimise the effects of RL-induced ROS production.

Both SL and RL-cells produced more ROS at treatment concentrations of 5-ALA than 0 mM groups. This suggests 5-ALA, without RL, causes enhanced oxidative stress and has the potential to cause dark toxicity at the concentrations used in this study. However, 0.5, 1, 2 and 4 mM RL-cells produced significantly more ROS than all other treatment groups in U2OS and TC71 cells. 5-ALA-PDT is therefore responsible for significantly enhancing ROS production and is more effective than RL, SL or 5-ALA alone in achieving this. HT1080s showed sensitivity to 2 mM 5-ALA, with SL and RL-cells producing more ROS than all other concentrations, the reason for this is not fully understood but it may relate to metabolic response or cellular saturation at this concentration. We acknowledge the skew of ROS data, but the use of Kruskal-Wallis assessed differences in group medians based on rank ordering, making it suitable for skewed data. As such, the results reflect differences in central tendency and distribution ranks across groups, rather than differences in means.

A further consideration with ROS studies was the change made from the normal protocol. Cells were not serum-starved overnight before the experiment, which may have enhanced PpIX efflux from the cells [55,56]. This was necessary to avoid false positive results of enhanced ROS production due to serum starvation rather than PDT [57]. However, cells were still treated with 5-ALA that had been diluted in SFM to maintain a minimal level of serum starvation required for some level of PpIX retention. This is acknowledged as a potential limitation as it means PpIX levels, therefore ROS levels, could differ between cells that were serum starved overnight (uptake studies) and cells that were not (ROS studies). However, protocols were consistent between all treatment groups and cell lines in ROS studies; those treated with PDT still showed consistently higher ROS levels than all other groups. Future experimentation would include controls to assess 5-ALA uptake under both conditions to facilitate cross-interpretation.

In line with the above points and findings of other studies, viability results showed 5-ALA-PDT effectively induced cytotoxicity in bone sarcoma cell lines [39,40,53]. Analysis of raw data showed, by 72 h, all 5-ALA-PDT cells had significantly lower absorbance values than 0 mM SL, 0 mM RL and all treatment concentrations of SL-cells. This indicates it is PDT, not RL, SL or 5-ALA independently, that causes reduction in cell viability and importantly, demonstrates lack of RL or dark-toxicity in any cells at 72 h. Use of raw absorbance data was necessary to deduce that the combination of 5-ALA and RL, that is PDT, is what elicits a cytotoxic effect and independently they have negligible effects. However, variation in raw data, consistent within repeats, but different between the three biological repeats, is not accounted for or standardised by the use of the constant in the viability equation. Viability data has been handled in a way that makes all repeats more representative of true viability in comparison to the growth of the 0 mM control in any given repeat, rather than raw data where variation exists for reasons outside of the effects of PDT. This is the reason all treatment concentrations were analysed using viability data and is a potential limitation of raw data analysis.

Viability results showed, at all time points, there was significantly more toxicity in RL-cells than SL-cells. By 24 h, all concentrations of RL-cells had significantly lower viability than all concentrations of SL-cells in every cell line. The only additional significant result at 72 h was 4 mM SL showing dark toxicity, in comparison to 0.5 and 1 mM SL-cells, in HT1080 and TC71 cell lines, respectively. 4 and 2 mM SL-cells frequently experienced some level of dark toxicity compared to 0, 0.5 and 1 mM treated cells. Dark toxicity at these higher concentrations, with largely insignificant differences in viability reduction in RL-cells, emphasises the need for down-titration and formal dose-response analysis in future experimentation, likely using a maximum dose of 2 mM and minimum doses smaller than 0.5 mM.

We acknowledge that the current study is limited to assessments of 5-ALA uptake, ROS production and cell viability. The primary aim of this work was to serve as a pilot investigation to establish the feasibility and potential of 5-ALA-PDT in sarcoma. The findings reported here provide a foundation for future, more comprehensive work that should incorporate investigations into the mechanisms of cell death, migration assays and chorioallantoic membrane assays to better characterise the effects of 5-ALA-PDT and further support its potential as an adjuvant treatment for sarcoma.

As with any therapeutic approach, PDT comes with certain constraints. Substantial consideration must be given to potential adverse effects and ways of mitigating these. Short-term adverse effects include local erythema, oedema and, the principal adverse effect, pain [71], usually successfully managed conservatively [72]. However, this is usually seen when PDT is used as an independent treatment. In the proposed use of intra-operative PDT, pain is unlikely to be a significant adverse effect due to the use of a general anaesthetic. Treating wound beds with PDT in sarcoma may cause vascular damage and impaired wound healing with long-term complications. Further to this, processes related to aging, malignancy and neurodegenerative diseases show ROS involvement [73], potentially severe complications that require randomised controlled trials and long-term prospective studies to be properly investigated. The long-term goal of this research would be to achieve standardization of 5-ALA-PDT in sarcoma, allowing for safe, efficacious regimes with predictable adverse effects.

## 5 Conclusion

The occurrence of three fundamental PDT events has been evidenced by this study: cellular 5-ALA uptake, significantly increased ROS production and significant cytotoxicity, following treatment with 5-ALA-PDT in bone sarcoma cells. This study provides preliminary *in vitro* results, but further research is necessary to optimise 5-ALA concentrations, radiation parameters and establish a control cell line, leading to the most desired outcomes and success in clinical practice. For bone sarcoma, 5-ALA-PDT has the potential to become an effective therapeutic strategy and, when used in combination with FGS and surgical resection, could be capable of lowering LR rates and improving long-term outcomes in bone sarcoma patients.

## Data Availability

Further data that that support the findings of this study are available from the Corresponding Author, [Rebecca H. Maggs], upon reasonable request.

## Abbreviations

**Abbreviation/Symbol**: **Meaning**
±: Plus-Minus
~: Approximately
°C: Degrees Celsius
μg/mL: Micrograms Per Millilitre
μL/well: Microlitres Per Well
μL: Microlitre
μm: Micrometre
μM: Micromolar
5-ALA: 5-Aminolevulinic Acid
5-ALA-PDT: 5-Aminolevulinic Acid-Induced Photodynamic Therapy
SL-cells: Sham Red Light Treated Cells
ANOVA: Analysis of Variance
ATCC: American Type Culture Collection
Cells/well: Cells per Well
CCK-8: Cell Counting Kit-8
CI: Confidence Interval
CM: Culture Medium
DAPI: 4^′^,6-diamidino-2-phenylindole
DNA: Deoxyribonucleic Acid
EDTA: Ethylenediaminetetraacetic Acid
FACS: Fluorescence-Activated Cell Sorting
FB: Flow Buffer
FBS: Foetal Bovine Serum
FDA: Food and Drug Administration
FGS: Fluorescence-Guided Surgery
FI: Fluorescence Intensity
h: Hours
IQR: Interquartile Range
J/cm^2^: Joules Per Centimetre Squared
LED: Light-Emitting Diode
LR: Local Recurrence
MFI: Median Fluorescence Intensity
mins: Minutes
mL: Millilitre
mM: Millimolar
mW: Milliwatts
mW/cm^2^: Milliwatts Per Centimetre Squared
nm: Nanometre
PBS: Phosphate Buffered Saline
PDT: Photodynamic Therapy
PpIX: Protoporphyrin IX
PS: Photosensitizer
RL: Red Light
RL-cells: Red Light Treated Cells
ROS: Reactive Oxygen Species
RPMI: Rosewell Park Memorial Institute
secs: Seconds
SD: Standard Deviation
SFM: Serum-Free Medium
SL: Sham Red Light
U/mL: Units Per Millilitre
UK: United Kingdom
VS: Versus
WHO: World Health Organisation

## Appendix A

Raw absorbance data (measured as optical density) obtained from CCK-8 assays are presented to provide the unprocessed measurements underlying the normalised results shown in the main text (Figs. 7–9). Data are shown for 0, 0.5, 1 and 2 mM concentrations for HT1080 (Fig. A1a–c), U2OS (Fig. A2a–c), and TC71 (Fig. A3a–c) cell lines. The main text includes optical density data for 4 mM treatment groups (Fig. 6a–c). Optical density readings for each concentration were recorded directly from the plate reader, with background correction performed using blank wells that contained fibronectin only. These raw data illustrate the variability across replicates and experimental conditions prior to normalisation against the 0 mM control. The data presented here uses Welch’s ANOVA and post-hoc Dunnett’s T3 multiple comparison test to investigate significant differences between the treatment group and the same concentration of SL-cells, 0 mM SL-cells and 0 mM RL-cells. By 72 h, raw absorbance data in the treatment group was significantly lower than the same concentration of SL-cells, 0 mM SL-cells and 0 mM RL-cells (*p* < 0.0001).
